# Acircularity and circularity indexes of the foveal avascular zone in high myopia

**DOI:** 10.1038/s41598-021-96304-9

**Published:** 2021-08-19

**Authors:** Helong Piao, Yue Guo, Haowei Zhang, Mi Sun Sung, Sang Woo Park

**Affiliations:** 1 Department of Ophthalmology and Research Institute of Medical Sciences, Chonnam National University Medical School and Hospital, 42 Jebong-ro, Dong-gu, Gwangju, 61469 South Korea; 2 Department of Ophthalmology, Affiliated Hospital of Chengdu University, Chengdu University, Chengdu, Sichuan China; 3 Eye Hospital and School of Ophthalmology and Optometry, Wenzhou Medical University, Wenzhou, Zhejiang China

**Keywords:** Biomarkers, Optics and photonics

## Abstract

This study explored the association between foveal avascular zone (FAZ) parameters and high myopia using optical coherence tomography angiography. We divided 106 eyes of 106 patients into quartiles based on the axial length. The upper quartile was then defined as the high myopia group (n = 27), while the lower quartile was the non-high myopia group (n = 26). The areas and minor axis lengths of superficial and deep FAZ, the perimeters and major axis lengths of deep FAZ were significantly larger in eyes with high myopia than in eyes with non-high myopia (*P* < 0.05). Inversely, the subfoveal choroidal thickness was significantly thinner in eyes with high myopia than in those with non-high myopia. Linear regression analyses showed that no significant correlation was observed between FAZ areas and acircularity and circularity indexes of FAZ in non-high myopia group. Conversely, FAZ areas strongly correlated with acircularity and circularity indexes of FAZ in high myopia group. We found that an increase in the FAZ area in highly myopic eyes was accompanied by a significant variation in FAZ acircularity and circularity indexes. Further research should address whether these findings are associated with future disease development in highly myopic eyes.

## Introduction

Myopization, especially high myopization, is a complex pathological process involving the whole eyeball. It is characterized by axial elongation, which can cause retinal detachment, choroid neovascularization, macular hemorrhage, and retinal ischemia, all of which lead to visual dysfunction^1–3^. One meta-analysis suggested that nearly half of the world’s population will be myopic by 2050, with up to 10% showing high myopia^4^. With the increased incidence of myopia, more knowledge about myopia progression is necessary. For several decades, many studies have focused on the pathological changes of myopia, including the effects of axial elongation on the chorioretinal thickness and retinal vasculature^5–9^. However, the pathological processes underlying myopia development remain unclear.

Recently, optical coherence tomography angiography (OCTA) has been commonly used in the clinical practice. It allows clinicians to view the various layers of the retinal and choroidal microvasculature non-invasively. Since many eye diseases involve the loss of retinal vessels, ophthalmologists could diagnose and manage patients using OCTA monitoring. One recent study showed that even when the OCT-measured parameters reach floor level, OCTA-measured vessel density is still present a promising indicator for monitoring progress in advanced glaucoma^10^. It follows that OCTA may possess better discriminatory power than OCT.

The foveal avascular zone (FAZ) is an important measurement parameter in OCTA. It is a capillary dropout zone in the macula and has the highest density of cone photoreceptors^5^. The FAZ is surrounded by an interconnecting capillary network derived from branches of the central retinal artery (CRA), which branches out on the retinal nerve fiber layer (RNFL) level to form the superficial plexus within the ganglion cell layer. Deeper branches reaching into the inner nuclear layer form the deep plexus^11^. Therefore, the FAZ reflects alterations in the central retinal microvasculature and is related to central visual function^12^. Several clinical elements can influence FAZ parameters, including diabetic retinopathy (DR), axial length, sex, and glaucoma^12–16^. Kwon et al. reported an increase in FAZ area and a decrease in the circularity index in glaucomatous eyes^12^. Krawitz et al. used the acircularity index and axial ratio of the FAZ to demonstrate that the FAZ shape was deformed in patients with DR^13^. He et al. and Cheng et al. showed that increased FAZ area and decreased vessel density were correlated with high myopia^14,15^. Interestingly, FAZ enlargement occurs in both glaucomatous and myopic eyes. Given that glaucoma is closely related to myopia, further research should address how the FAZ varies among myopic individuals.

Since myopia progression mainly affects the posterior pole of the retina through axial elongation, highly myopic eyes may show some characteristic changes in the FAZ. Accordingly, in the present study, we measured the acircularity index, circularity index, and other biomarkers of the FAZ to quantify the shape of the FAZ and determine whether FAZ deformation was significantly correlated with high myopia.

## Results

### Clinical features and parameters of the subjects

A total of 106 eyes of 106 subjects with myopia were enrolled. The general characteristics of the study subjects are listed in Table 1. The mean axial length was 25.9 ± 1.4 mm (range: 22.0–28.1 mm); mean spherical equivalent, − 5.4 ± 2.9 diopters (range: − 12.125 to − 0.25 diopters); mean foveal thickness, 251.0 ± 19.8 μm (range: 200–297 μm); and subfoveal choroidal thickness, 258.4 ± 17.8 μm (range: 80 to 393 μm). Intraclass correlation coefficients (ICC) and associated 95% confidence intervals (CI) were calculated in SPSS. Inter-rater reliability for the all of FAZ measurements showed excellent reproducibility (ICC = 0.96, CI = 0.93–0.97 in superficial layer and ICC = 0.92, CI = 0.86–0.97 in deep layer).

**Table 1:** Demographic and ocular factors of study subjects.

	Number of subjects (n = 106)		
	Mean	SD	Range
Age (years)	23.8	4.2	18 to 34
Sex, male/female	74/32		
Axial length (mm)	25.9	1.4	22.1 to 28.1
IOP (mmHg)	14.2	2.6	10 to 21
Spherical equivalent (diopter)	− 5.4	2.9	− 12.125 to − 0.25
Central corneal thickness (μm)	555.5	31.0	487 to 632
Average keratometry (diopter)	42.4	2.3	38.2 to 47.2
Foveal thickness (μm)	251.0	19.8	200 to 297
Parafoveal thickness (μm)	313.4	15.2	268 to 356
Subfoveal choroidal thickness (μm)	258.4	17.8	80 to 393

*IOP* intraocular pressure.

### Ocular factors and FAZ parameters of OCTA

We grouped all subjects based on the method recommended by Joarder and Firozzaman^17^. We first defined the upper and lower quartiles (upper quartile: n = 27, cut-off value = 26.84 mm; lower quartile: n = 26, cut-off value = 25.02 mm). The remaining 53 subjects belonged to the second and third quartiles. After grouping, only those with high myopia were included in the upper quartile group, while none of those with high myopia were in the lower quartile group, in which all participants had low to moderate myopia. The results of the grouping and a comparison of FAZ parameters between the two groups are shown in Table 2. In the non-high myopia group, the mean axial length was 23.96 ± 0.94 mm (range: 22.09–25.02 mm) and the spherical equivalent was − 2.27 ± 1.58 diopters (range: − 5.25 to − 0.25 diopters), while in the high myopia group, the mean axial length was 27.42 ± 0.36 mm (range: 26.86–28.11 mm) and the spherical equivalent was − 8.12 ± 2.14 diopters (range: − 12.125 to − 6.25 diopters). The following measurements were significantly larger in eyes with high myopia than in those with non-high myopia: the areas of the superficial and deep FAZ, the perimeters of the deep FAZ, the major axis lengths of the deep FAZ, and the minor axis lengths of the superficial and deep FAZ (*P* < 0.05 in all cases). Inversely, the subfoveal choroidal thickness was significantly thinner in eyes with high myopia than in those with non-high myopia. No other parameter showed any significant difference between the groups (acircularity index, circularity index, and axial ratio of the superficial and deep FAZ). These results showed that significant differences occurred more often with the deep FAZ.

**Table 2:** Ocular factors and FAZ parameters of OCTA in non-high myopia (lower quartile) and high myopia (upper quartile) groups.

Variables	Non-high myopia (n = 26)	High myopia (n = 27)	*P* Value
Axial length (mm)	24.0 ± 0.9 (22.09 to 25.02)	27.4 ± 0.4 (26.86 to 28.11)	** < 0.001**
Spherical equivalent (diopter)	− 2.3 ± 1.6 (− 5.25 to − 0.25)	− 8.1 ± 2.1 (− 12.125 to − 6.25)	** < 0.001**
Central corneal thickness (μm)	539.65 ± 34.58 (490 to 594)	554.81 ± 32.71 (498 to 603)	0.908
Foveal thickness (μm)	247.73 ± 13.55 (214 to 267)	252.74 ± 22.13 (209 to 297)	0.200
Parafoveal thickness (μm)	315.19 ± 12.97 (296 to 342)	311.33 ± 13.49 (287 to 343)	0.323
Subfoveal choroidal thickness (μm)	317.88 ± 49.50 (182 to 393)	203.11 ± 90.54 (80 to 371)	** < 0.001**
Area of superficial FAZ (mm^2)^	0.3 ± 0.08 (0.18 to 0.52)	0.43 ± 0.22 (0.20 to 1.15)	**0.024**
Area of deep FAZ (mm^2^)	0.45 ± 0.12 (0.23 to 0.68)	0.66 ± 0.25 (0.33 to 1.36)	** < 0.001**
Perimeter of superficial FAZ (mm)	2.9 ± 0.46 (2.05 to 4.08)	3.47 ± 1.41 (2.06 to 8.60)	0.122
Perimeter of deep FAZ (mm)	3.96 ± 0.71 (2.79 to 5.62)	4.87 ± 1.41 (2.82 to 8.93)	**0.005**
Circularity index of superficial FAZ	0.48 ± 0.10 (0.29 to 0.67)	0.48 ± 0.12 (0.20 to 0.67)	0.873
Circularity index of deep FAZ	0.37 ± 0.06 (0.23 to 0.46)	0.36 ± 0.09 (0.20 to 0.56)	0.423
Acircularity index of superficial FAZ	1.47 ± 0.17 (1.22 to 1.85)	1.49 ± 0.24 (1.22 to 2.27)	0.873
Acircularity index of deep FAZ	1.67 ± 0.17 (1.47 to 2.11)	1.69 ± 0.21 (1.33 to 2.26)	0.423
Axial ratio of superficial FAZ	1.24 ± 0.19 (1.03 to 1.92)	1.21 ± 0.20 (1.01 to 1.70)	0.310
Axial ratio of deep FAZ	1.18 ± 0.13 (1.01 to 1.48)	1.24 ± 0.17 (1.02 to 1.65)	0.179
Major axis length of superficial FAZ (mm)	0.78 ± 0.11 (0.55 to 1.01)	0.93 ± 0.34 (0.55 to 2.09)	0.065
Major axis length of deep FAZ (mm)	0.93 ± 0.15 (0.67 to 1.31)	1.13 ± 0.21 (0.82 to 1.71)	** < 0.001**
Minor axis length of superficial FAZ (mm)	0.64 ± 0.10 (0.44 to 0.87)	0.77 ± 0.26 (0.43 to 1.72)	**0.012**
Minor axis length of deep FAZ (mm)	0.79 ± 0.11 (0.60 to 1.02)	0.92 ± 0.15 (0.72 to 1.34)	**0.003**

*FAZ* foveal avascular zone.

Factors with statistical significance are shown in boldface.

### Associations of the FAZ parameters of OCTA with FAZ area

**Figure 1:**
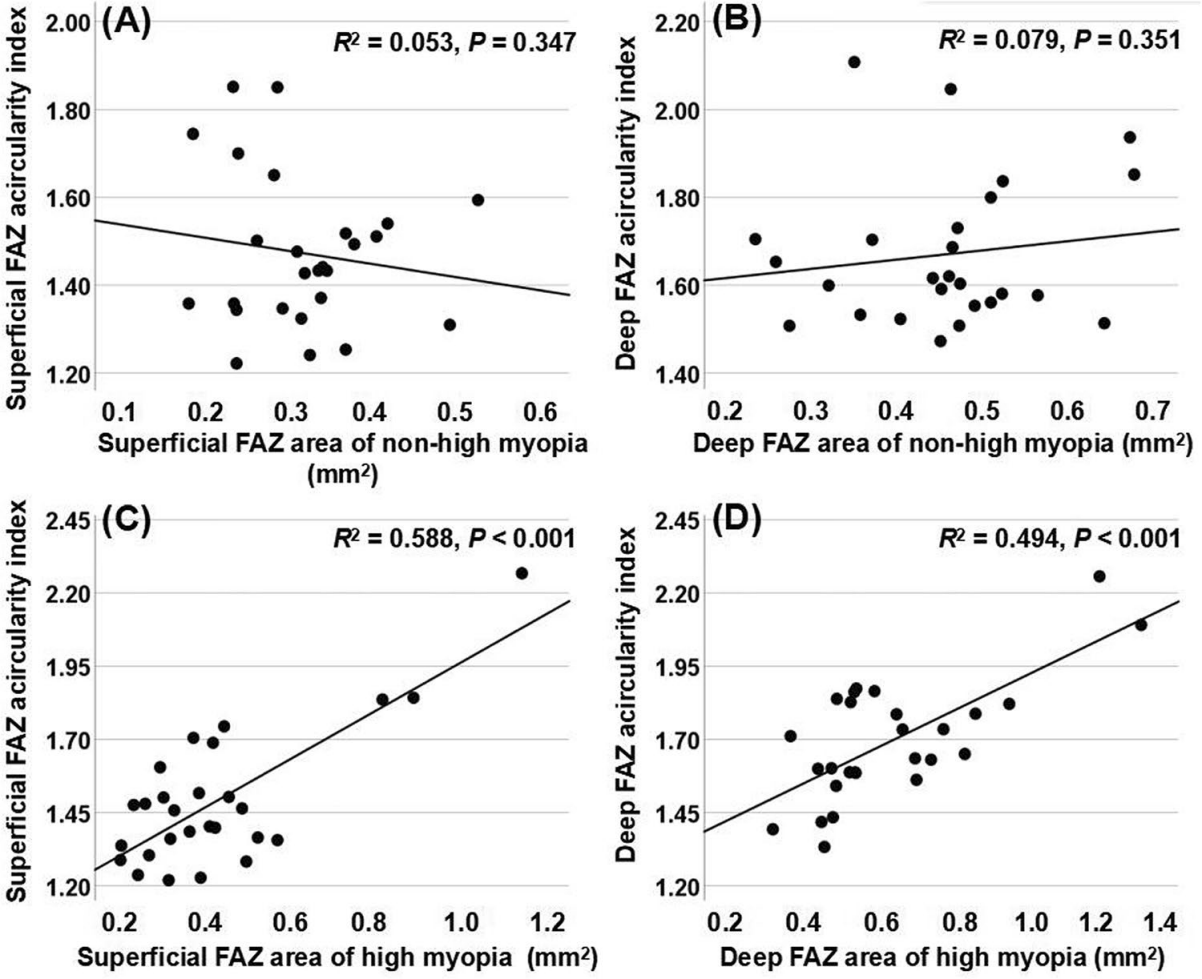
Scatter plots showing the association between the foveal avascular zone (FAZ) areas and acircularity index of FAZ. (**A**, **B**) The areas of the superficial and deep FAZ were not significantly correlated with the acircularity index in the non-high myopia group. (**C**, **D**) The areas of the superficial and deep FAZ were strongly associated with the acircularity index in the high myopia group.

Linear regression analysis adjusted for age and sex was conducted to examine whether the FAZ parameters of OCTA were correlated with FAZ area in the non-high myopia and high myopia groups. As shown in Table 3, the areas of the superficial and deep FAZ were not significantly correlated with the circularity index of the FAZ in the non-high myopia group. Conversely, the areas of the superficial and deep FAZ were significantly negatively correlated with circularity index (*P* = 0.001 in both layers) of the FAZ in the high myopia group. Likewise, the areas of the superficial and deep FAZ were not significantly correlated with the acircularity index of the FAZ in the non-high myopia group (Fig. 1A, B), but the areas of the superficial and deep FAZ were significantly positively correlated with acircularity index of the FAZ in the high myopia group (*P* < 0.001 in both layers) (Fig. 1C, D). In both groups, the areas of the superficial and deep FAZ were significantly positively correlated with the perimeters of the FAZ (*P* < 0.001 in both layers). Only the areas of the superficial FAZ in the non-high myopia group were significantly correlated with the axial ratio of the FAZ (*P* = 0.011). In addition, the areas of the superficial FAZ in the non-high myopia group were weakly correlated with the major axis lengths of the FAZ (*P* = 0.024), while other major and minor axis lengths of the FAZ were strongly associated with the FAZ areas (*P* < 0.001 in both groups).

**Table 3:** Associations of the FAZ parameters of OCTA with FAZ area in non-high myopia (lower quartile) and high myopia (upper quartile) groups.

	Area of superficial FAZ			Area of deep FAZ		
	*R*^2^	Coefficient	*P* Value*	*R*^2^	Coefficient	*P* Value*
**Non-high myopia**						
Circularity index of FAZ	0.027	0.190	0.536	0.069	0.001	0.997
Acircularity index of FAZ	0.053	− 0.478	0.347	0.079	− 0.040	0.951
Perimeter of FAZ	0.534	3.787	** < 0.001**	0.704	4.553	** < 0.001**
Axial ratio of FAZ	0.269	− 1.339	**0.011**	0.013	0.129	0.659
Major axis length of FAZ	0.406	0.580	**0.024**	0.697	1.074	** < 0.001**
Minor axis length of FAZ	0.818	1.086	** < 0.001**	0.754	0.853	** < 0.001**
**High myopia**						
Circularity index of FAZ	0.427	− 0.352	**0.001**	0.409	− 0.223	**0.001**
Acirculariy index of FAZ	0.588	0.814	** < 0.001**	0.494	0.595	** < 0.001**
Perimeter of FAZ	0.931	6.243	** < 0.001**	0.915	5.516	** < 0.001**
Axial ratio of FAZ	0.094	0.186	0.336	0.254	0.065	0.600
Major axis length of FAZ	0.892	1.458	** < 0.001**	0.708	0.707	** < 0.001**
Minor axis length of FAZ	0.764	1.030	** < 0.001**	0.805	0.520	** < 0.001**

*FAZ* foveal avascular zone.

*P value adjusted for age and sex.

Factors with statistical significance are shown in boldface.

## Discussion

In this study, we utilized OCTA to analyze the macular microvasculature and further explore the morphological changes of FAZ. We found that several FAZ parameters were larger in highly myopic eyes than in non-highly myopic eyes, and that the differences were even more pronounced in the deep FAZ layer. Moreover, the acircularity and circularity indexes of the FAZ were significantly correlated with FAZ enlargement in highly myopic eyes, whereas no significant relationships were found between these parameters in the non-highly myopic group. To our knowledge, this was the first report to determine FAZ deformation in high myopia.

Several reports have investigated the FAZ in myopic eyes^5,14–16,18,19^. Sung et al. reported lower peripapillary vessel density and larger superficial and deep FAZ areas in highly myopic eyes, as elucidated using OCTA^5^. He et al. also demonstrated that reduced radial peripapillary capillary, deep parafoveal vessel density, and enlarged FAZ area occurred in high myopia^14^. Similarly, Cheng et al. showed that increased FAZ area and decreased vessel density in the superficial and deep layers were correlated with axial elongation^15^. All these studies reported that FAZ enlargement was accompanied by a reduction of retinal vessel density in the macular region, indicating that the size of the FAZ indirectly reflects variations in retinal perfusion. Although we did not measure vessel density in the present study, our results are in accordance with those of the previous reports.

The mechanism of FAZ enlargement in myopic eyes remains unclear, although some hypotheses have been offered. First, retinal vascular trunk dragging during myopia progression may decrease retinal blood flow and further result in variation in the FAZ size. Two previous studies reported that the FAZ areas were significantly associated with optic nerve head tilt, which occurs during axial elongation^5,14^. Second, in eyes with axial elongation, overall macular thinning may cause reduced oxygen consumption, resulting in decreased retinal blood flow and increased FAZ area^20,21^. In the current study, the FAZ areas in highly myopic eyes were significantly larger than those in non-highly myopic eyes, and the difference was more significant in the deep FAZ layers, suggesting that the deep capillary plexus is more susceptible to myopia-related changes, possible because the arterial blood supply differs between the superficial and deep capillary plexuses. The oxygen and nutrition demand of the superficial retina is met by the central retinal artery, while that of the deep retina is met by the choroidal vascular system^22^. In our study, the subfoveal choroidal thickness was significantly thinner in eyes with high myopia than in those without high myopia. This result is in line with the study by Al-Sheikh et al.^23^ who reported that choroidal thinning is more prominent in highly myopic eyes. Thus, the FAZ enlargement might be more pronounced in the deep retinal layer.

Numerous studies have researched FAZ deformation, but none have explored the morphological changes in the FAZ in high myopia. We speculated that either or both of the following mechanisms influence FAZ morphology: First, the irregular reduction in macular blood perfusion caused by the retinal vascular trunk dragging, with consequent FAZ deformation and enlargement during axial elongation. Second, as the retinal vascular trunk was dragged nasally in highly myopic eyes, the FAZ in the macula may have been distorted towards the ONH, leading to FAZ deformation. Recent prospective observational study on progressive myopia demonstrated that the central vascular trunk in the lamina cribrosa was dragged nasally during axial elongation^24^. Therefore, depending on geometrical change in the FAZ, it may be possible to identify myopia severity and macular perfusion status. Future longitudinal study should verify this.

**Figure 2:**
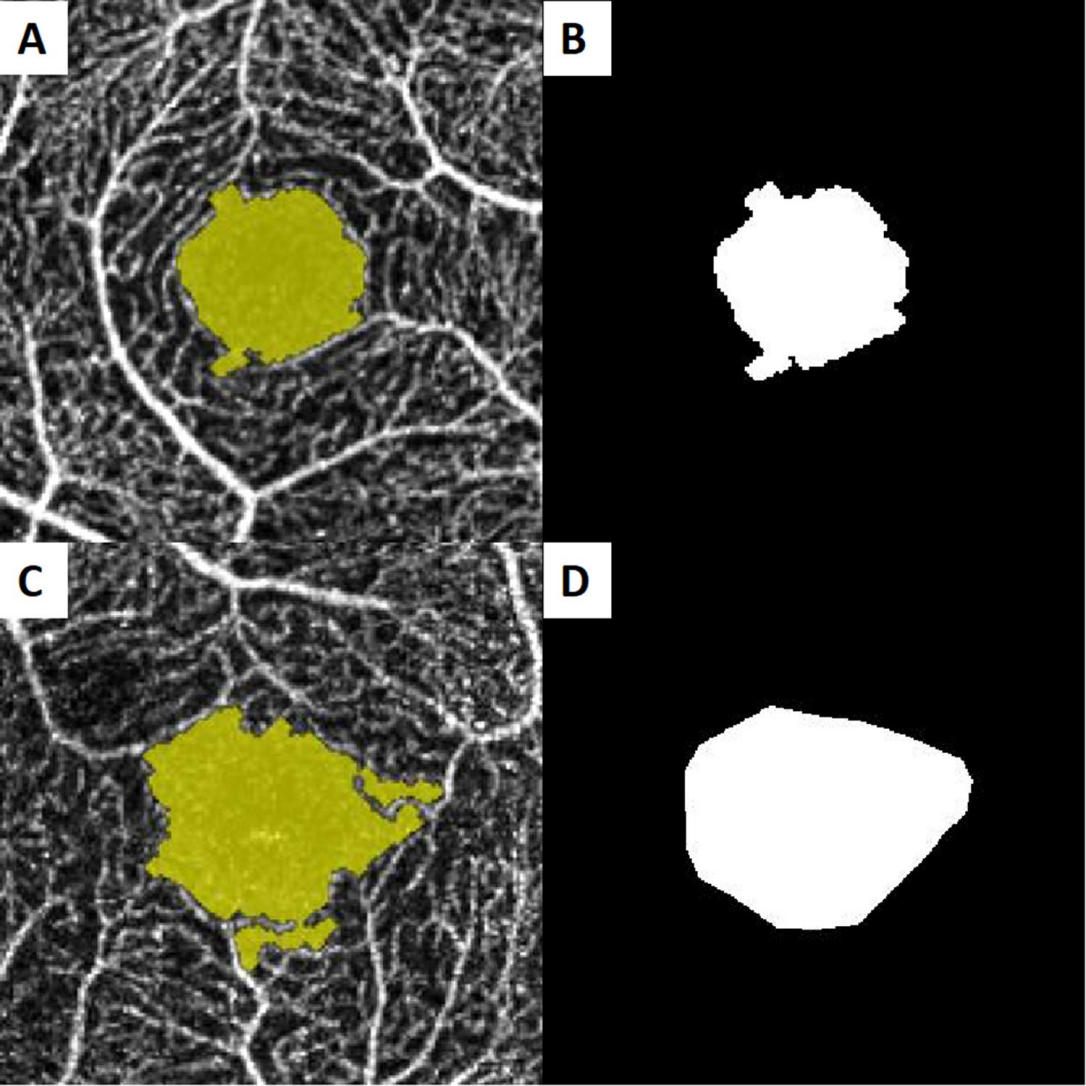
(**A**, **B**) Optical coherence tomography angiogram of the foveal avascular zone (FAZ) of non-high myopia in superficial layer. (**C**, **D**) Optical coherence tomography angiogram of the FAZ of high myopia in superficial layer.

So far, to our knowledge, there has been no study regarding the FAZ circularity index of high myopia and non-high myopia. However, a number of papers have been reported that FAZ circularity was decreased after suffering a period of pathophysiological process in several disease status^25–29^. They showed the FAZ shape relatively circular initially, and evolved from circular to acircular during disease progression. Figure 2A, B. are the scan images from a non-highly myopic eye. Because the eye was less affected by aforementioned two mechanisms, the shape was approach to circular. In contrast, Fig. 2C, D. are the representative images of highly myopic eye, and we can see the increase in FAZ area and decrease in circularity index. We infer that FAZ enlargement in non-high myopia is attribute to individual variation and not the result of authentic FAZ enlargement, hence the significant change of circularity index was not found in this group. As previous study showed that the circularity index is proportional to the area and inversely proportional to the square of the perimeter. In DR, the FAZ has a higher amount of irregular outlines, thus increasing the perimeter for a given surface value compared with a healthy control^26^. Likewise, the high myopization may not only cause an increase of the FAZ area, but also higher amount of irregular outlines and longer perimeter, ultimately lead to circularity index decrease.

Of note, we found that the FAZ areas were not significantly associated with the acircularity or circularity index in non-highly myopic eyes. However, they were positively correlated with acircularity index and negatively correlated with circularity index in highly myopic eyes, suggesting that the FAZ area becomes larger and more acircular as myopia progresses. Variations in the FAZ acircularity/circularity indexes and FAZ size may have multiple clinical significances. Choi et al. reported that decreased FAZ circularity index may represent a disruption of the parafoveal capillary network in patients with glaucoma^25^. Kwon et al. showed that a decreased FAZ circularity index was significantly associated with central visual field defect, and that the FAZ size was significantly associated with the severity of central visual field defect in glaucomatous eyes^26^. Kim et al. also showed that decreased FAZ circularity is a good indicator of vascular dropout, and that it is associated with disease progression in vascular maculopathy^27^. Another study found a significant difference in acircularity index between controls and non-proliferative DR. This result could inform objective staging of the disease^13^, before the disease has progressed significantly, the retinal microvascular system in myopic eyes has already suffered structural damage, and discernible microvascular changes are visible in the FAZ. Given that FAZ acircularity is associated with visual field defects and macular vascular dropout, our results might explain the reason for the high prevalence of central scotoma in myopic glaucoma^30^.

Unlike other indicators such as area, perimeter, and length, the acircularity index can quantify FAZ geometry without the need for axial length measurements to correct for retinal magnification^13^. It follows that the acircularity index (including circularity index) could serve as indicators to predict pathological myopic changes in advance. Long-term monitoring of FAZ evolution using the acircularity and circularity indexes may be necessary to substantiate the clinical value of these metrics, which are not influenced by ocular magnification correction.

The limitations of the current study were as follows: The study was retrospective, and to evaluate the specific influence of axial myopia, we set further criteria, which resulted in a small sample size. A prospective big data study is needed to improve data accuracy. Furthermore, we did not measure the retinal thickness in detail. One previous study demonstrated that early foveal microcirculatory alterations in diabetic eyes were related to macular ganglion cell/inner plexiform layer thickness, regardless of the presence of DR^27^. Intraretinal thickness may therefore be related to FAZ deformation in myopic eyes. Future studies should investigate this relationship further to deepen the current understanding of myopia progression.

In conclusion, we observed FAZ enlargement in highly myopic eyes, especially in the deep retinal layer. This enlargement accompanied changes in the acircularity and circularity indexes of the FAZ in highly myopic eyes. Further research should address whether these findings are associated with future disease development in highly myopic eyes.

## Methods

### Subjects

This retrospective, cross-sectional study was conducted according to the tenets of the Declaration of Helsinki and was approved by the Institutional Review Board of Chonnam National University Hospital. The participants were informed of the study objectives and written informed consent was obtained from all participants.

Healthy myopic volunteers were recruited consecutively from July 1, 2016 to September 31, 2016. A detailed medical history was recorded for each subject. All subjects underwent complete ophthalmic examination, including best-corrected visual acuity (BCVA), intraocular pressure (IOP) using Goldmann applanation tonometry, slit-lamp examination, refractive error assessment, fundus examination using color stereoscopic disc photography and red-free RNFL fundus photography, and Swedish Interactive Threshold Algorithm standard 30–2 perimetry using a Humphrey Field Analyzer (Carl Zeiss Meditec Inc., Dublin, California, USA). All IOP measurements were recorded between 4:00 PM and 7:00 PM. Axial length and central corneal thickness were measured using optical low-coherence reflectometry (Lenstar; Haag-Streit AG, Koeniz, Switzerland).

All volunteers were required to meet the following inclusion criteria: (1) age between 18 and 40 years; (2) astigmatism within ± 2 diopters; (3) BCVA of 20/25 or better; (4) IOP ≤ 21 mmHg; (5) normal optic nerve head with non-glaucomatous changes on stereoscopic photographs, including an intact neuroretinal rim without thinning, pit, or localized pallor; (6) absence of RNFL abnormalities on red-free fundus photographs; and (7) normal visual field results in both eyes. The exclusion criteria included (1) history of glaucoma or intraocular surgery; (2) IOP > 21 mmHg; (3) myopic degeneration, including peripheral retinal tears, intrachoroidal cavitations, or choroidal neovascularization; (4) history of systemic disease that could affect the eyes, such as diabetes mellitus, (5) other evidence of retinal pathology or opaque media. For cases in which both eyes of a subject met the inclusion criteria, one eye was chosen randomly.

### Spectral-domain optical coherence tomography

All subjects underwent macular imaging with Avanti spectral-domain OCT (RTVue-XR Avanti; Optovue, Fremont, California, USA). The fovea was defined as the area within the central 1-mm ring and the parafoveal area within the central 3-mm ring of the Early Treatment Diabetic Retinopathy Study grid. The distance between the internal limiting membrane (ILM) and the middle of the retinal pigment epithelium (RPE) as the full retinal layer thickness. Subfoveal choroidal thickness was evaluated by the enhanced depth imaging (EDI) technique of the Avanti spectral-domain OCT device. From the 12-mm-length EDI scan running through the fovea, vertical distance from the hyper-scattering outer border of the RPE to the inner border of the sclera at the center of the fovea was measured and it was defined as subfoveal choroidal thickness.

### Spectral-domain optical coherence tomography angiography

The spectral-domain OCT instrument used was the RTVue Avanti with prototype AngioVue® OCTA software (Optovue, Inc., Fremont, CA). This instrument operates at ~ 840 nm wavelength and conducts 70,000 A-scans per second to acquire OCTA volumes consisting of two repeated B-scans from 304 sequential, uniformly spaced locations. Each B-scan consists of 304 A-scans, giving a total of 2 × 304 × 304 A-scans per acquisition, with a total acquisition time of approximately 3 s and an axial optical resolution of ~ 5 μm. Macular 3 × 3-mm region scans centered on the fovea were captured from each subject. OCTA perfusion maps were generated using the split-spectrum amplitude decorrelation angiography (SSADA) algorithm, as previously described^31^. The Optovue software built into the Avanti OCT automatically segmented the inner retinal blood vessels into the superficial and deep capillary plexus layers. The boundaries of the superficial capillary plexus extended from 3 mm below the ILM to 15 mm below the inner plexiform layer. The deep capillary plexus extended from 15 to 70 mm below the IPL.

### FAZ measurements

**Figure 3:**
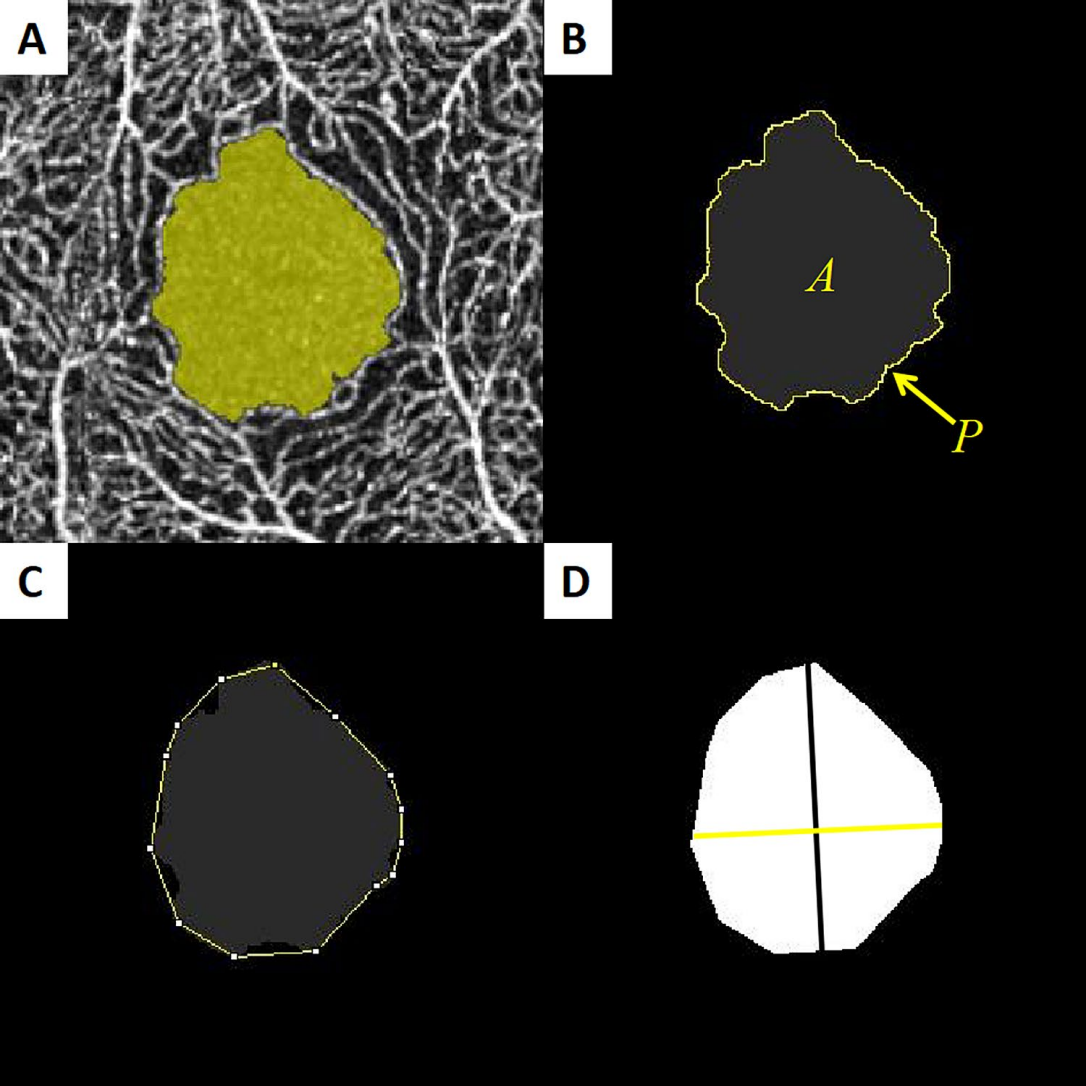
(**A**) Optical coherence tomography angiogram of the foveal avascular zone (FAZ) in superficial layer. (**B**) The FAZ was separated from the background image and the area (*A*) and perimeter (*P*) were automatically measured using the ImageJ software. (**C**) Outline was manually fit using the ImageJ software. (**D**) The major axis (black line) and minor axis (yellow line) lengths were also manually calculated.

**Figure 4:**
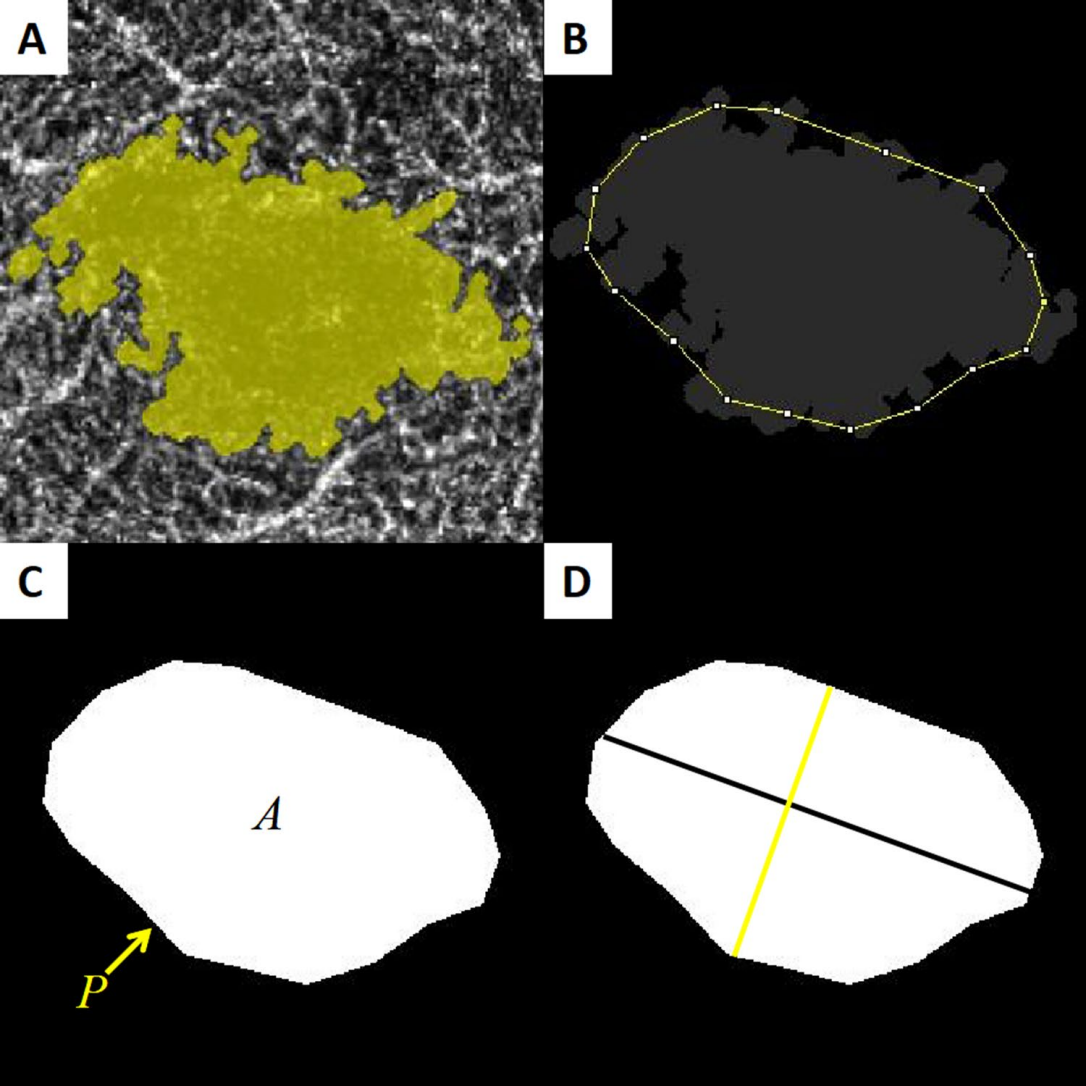
(**A**) Optical coherence tomography angiogram of the foveal avascular zone (FAZ) in deep layer. (**B**) An irregular outline was manually fit using the ImageJ software. (**C**) The FAZ area (*A*) and perimeter (*P*) were automatically measured using the ImageJ software after outlining the FAZ. (**D**) The major axis (black line) and minor axis (yellow line) lengths were manually calculated.

To investigate the morphological changes in the FAZ, the following measurements of FAZ superficial and deep layers were made, as described in detail elsewhere: FAZ area, FAZ perimeter, acircularity index, circularity index, axial ratio, major axis length, and minor axis length^13,32^. In the superficial layer, the area and perimeter length of relatively regular FAZ could be measured automatically with ImageJ software 1.52a (National Institutes of Health, USA), then we manually measured the major axis length and minor axis length after outlining FAZ manually using ImageJ software (Fig. 3). In the deep layer, we firstly outlined the irregular FAZ manually, then measured the area and perimeter length automatically and the major axis length and minor axis length manually using ImageJ software (Fig. 4). If there was a very irregular FAZ in the superficial layer, we would also measure it according to the method of measuring deep layer. Each OCTA image contains 605 × 605 pixel TIF was used for analysis with ImageJ software. Littman and the modified Bennett formulas were used to correct ocular magnification induced by axial variation on the area of FAZ^33^. A perfectly circular FAZ has an acircularity index equal to 1.0, with deviations from a circular shape leading to an increase in this metric. Acircularity is defined by the following equation:

$$ {\text{Acircularity }} = {\text{ perimeter}}\,{\text{of}}\,{\text{FAZ}}/{\text{perimeter}}\,{\text{of}}\,{\text{the}}\,{\text{circle}}\,{\text{with}}\,{\text{equal}}\,{\text{area}} $$

Circularity is a shape descriptor that can mathematically indicate the degree of similarity of the FAZ to a perfect circle. A value of 1.0 designates a perfect circle; as the circularity value decreases, the shape is increasingly less circular. Circularity is defined by the following equation:

$$ {\text{Circularity }} = \, 4\pi \, \times {\text{ area }}/{\text{ perimeter}}^{2} $$

The following parameters were determined from a best-fit ellipse of the FAZ: the length of the major and minor axes, and the axial ratio, which is calculated using the following equation:

$$ {\text{Axial}}\,{\text{ratio }} = {\text{ major}}\,{\text{axis}}\,{\text{length}}\,{\text{of}}\,{\text{FAZ}}/{\text{minor}}\,{\text{axis}}\,{\text{length}}\,{\text{of}}\,{\text{FAZ}} $$

To decrease the measurement error, the average data from two independent examiners (H.P. and H.Z.) were used statistically in this study.

### Statistical analysis

IBM SPSS Statistics for Windows, Version 25.0 (IBM Corp., Armonk, NY, USA) was used for statistical analysis. The inter-observer agreement of the FAZ parameters was evaluated by calculating the ICC. The Mann–Whitney U test was used to compare the FAZ parameters between non-high myopia and high myopia groups. Linear regression analysis was used to investigate whether the areas of the superficial and deep FAZ were correlated with other FAZ parameters in each group. *P*-values < 0.05 were considered statistically significant.

## Data Availability

The datasets generated during the current study are available from the corresponding author on reasonable request.
